# FluTE, a Publicly Available Stochastic Influenza Epidemic Simulation Model

**DOI:** 10.1371/journal.pcbi.1000656

**Published:** 2010-01-29

**Authors:** Dennis L. Chao, M. Elizabeth Halloran, Valerie J. Obenchain, Ira M. Longini

**Affiliations:** 1 Center for Statistics and Quantitative Infectious Diseases/Fred Hutchinson Cancer Research Center, Seattle, Washington, United States of America; 2 Department of Biostatistics, School of Public Health/University of Washington, Seattle, Washington, United States of America; University of Oxford, United Kingdom

## Abstract

Mathematical and computer models of epidemics have contributed to our understanding of the spread of infectious disease and the measures needed to contain or mitigate them. To help prepare for future influenza seasonal epidemics or pandemics, we developed a new stochastic model of the spread of influenza across a large population. Individuals in this model have realistic social contact networks, and transmission and infections are based on the current state of knowledge of the natural history of influenza. The model has been calibrated so that outcomes are consistent with the 1957/1958 Asian A(H2N2) and 2009 pandemic A(H1N1) influenza viruses. We present examples of how this model can be used to study the dynamics of influenza epidemics in the United States and simulate how to mitigate or delay them using pharmaceutical interventions and social distancing measures. Computer simulation models play an essential role in informing public policy and evaluating pandemic preparedness plans. We have made the source code of this model publicly available to encourage its use and further development.

## Author Summary

Computer simulations can provide valuable information to communities preparing for epidemics. These simulations can be used to investigate the effectiveness of various intervention strategies in reducing or delaying the peak of an epidemic. We have made a detailed influenza epidemic simulator for the United States publicly available so that others may use the software to inform public policy or adapt it to suit their needs.

## Introduction

Mathematical and computer models of epidemics have contributed to our understanding of the spread of infectious disease and the measures needed to contain or mitigate them [1]–[9]. Detailed computer simulations will play an important role in evaluating containment and mitigation strategies for future epidemics [8]. Although many simulation models have been described in the literature, few are publicly available. Releasing the source code of models would allow others to evaluate the quality of the simulation, replicate results, and alter and improve the model.

We have released the source code for a new stochastic model of influenza epidemics, FluTE. FluTE is an individual-based model capable of simulating the spread of influenza across major metropolitan areas or the continental United States. The model's structure is based on previously published work [3],[6], but FluTE incorporates a more sophisticated natural history of influenza, more realistic intervention strategies, and can run on a personal computer. Here, we describe the new model and illustrate how it can be used to study the dynamics of an epidemic and to investigate the population-level effects of interventions.

## Model

FluTE is an individual-based simulation model of influenza epidemics. In this section, we describe the model's community structure, natural history of influenza, and simulated interventions. Briefly, all individuals in the model are members of social mixing groups, within which influenza is transmitted by random mixing. The model can simulate several intervention strategies, and these can either change the transmission characteristics of influenza (e.g., vaccination) or change the contact probabilities between individuals (e.g., social distancing). Interventions can occur before the epidemic or in response to an ongoing epidemic.

### Community structure and social contacts

The simulation creates synthetic populations based on typical American communities. The population is divided into census tracts, and each tract is subdivided into communities of 500–3000 individuals based on earlier models [6],[10]. Each community is populated by randomly generated households of size 1–7 using the US-wide family size distribution from the 2000 Census (Table 1). The household is the closest social mixing group, within which contacts between individuals occur most frequently and thus influenza is transmitted most often. The population is organized as a hierarchy of increasingly large but less intimate mixing groups, from the household cluster (sets of four socially close households), neighborhoods (1/4 of a community), and the community. Although the model results are not sensitive to the exact size of these groups, including such groups creates a realistic contact network for disease transmission [11]. At night, everyone can make contact with other individuals in their families, household clusters, home neighborhoods, and home communities. In the daytime, individuals might interact with additional groups. During the day, most children attend school or a playgroup, where there is a relatively high probability of transmission. Preschool-age children usually belong to either a playgroup of four children or a neighborhood preschool, which typically has 14 students. Each community has mixing groups that represent two elementary schools, one middle school, and one high school, which typically have 79, 128, and 155 students, respectively.

**Table 1:** Frequency of household sizes.

*Frequency*	*Family size*
33%	single adult
34%	two people (two adults or a parent and child)
13%	two adults, one child
10%	two adults, two children
7%	two adults, three children
2%	two adults, four children
1%	two adults, five children

Data from [6].

Most working-age adults (about 72% of 19–64 year-olds) are employed. Employment rates are determined on a tract-by-tract basis using data from the US Census 2000's Summary File 3, table PCT35. Employed individuals often work outside of their home communities. Each employed individual is assigned to work in a destination census tract based on commuting data taken from Part 3 of the Census Transportation Planning Package (http://www.fhwa.dot.gov/ctpp/dataprod.htm), which provides information on the home and destination census tracts of workers in the United States. We eliminated commutes over 100 miles from the data as in [6] because many of these trips represent sporadic long-distance travel rather than daily commutes. Working individuals are assigned to communities and neighborhoods within their destination tracts to simulate casual community contacts during the day, and a work group of about 20 people to represent their close contacts at the workplace. Unemployed individuals remain in their home communities and do not have close daytime contacts except with members of their households who are not employed or enrolled in school.

Individuals can engage in short-term, long-distance domestic travel to represent vacations and other trips. Travel in our model is based on the implementation in [6], which uses data from the 1995 American Travel Survey data available from the U. S. Department of Transportation, Bureau of Transportation Statistics (http://www.bts.gov/publications/national_transportation_statistics/). Each day, an individual has a fixed probability of starting a trip based on an age-specific probability of traveling: 0.0023 for 0–4 year olds, 0.0023 for 5–18, 0.0050 for 19–29, 0.0053 for 30–64, and 0.0028 for 65 and older. The traveler will stay at the destination for 0–11 nights, with 23.9% of trips lasting for a single day (and no nights), 50.2% including 1–3 nights away, 18.5% including 4–7 nights away, and 7.4% for 8–11 nights. We do not include differences in travel frequency or duration during different times of the year (e.g., summer and holiday trips). The destination is a randomly selected census tract, in which a random community, neighborhood, and workplace (if the traveler is between 19 and 64 years old) are assigned to be the traveler's mixing groups. A random member of this community is assigned to be the traveler's contact person, and at night the traveler will behave as if he/she belongs to the contact's household, household cluster, and neighborhood. The traveler may withdraw to this household if ill. The exact implementation of short-term, long-distance travel is not important, but some long-distance travel is required in large populations for the epidemic to spread in a realistic manner. For simulations of smaller regions, such as a single county, there is no need to include long-distance travel.

New infected individuals are introduced to a simulation by infecting randomly selected people. This epidemic seeding process can occur once at the beginning of a simulation or daily. In addition, one can simulate an epidemic that is seeded from international travelers. In this scenario, randomly selected individuals in the counties with one of the United States' 15 busiest international airports are infected each day, proportional to the daily traffic of these airports (see Table 2).

**Table 2:** International traffic to the 15 US airports built into FluTE.

Airport	City	Passengers/year
JFK	New York, NY	21,842,544
LAX	Los Angeles, CA	17,019,166
MIA	Miami, FL	15,509,279
ORD	Chicago, IL	11375367
EWR	Newark, NJ	10,812,993
ATL	Atlanta, GA	9,166,055
SFO	San Francisco, CA	8,648,219
IAH	Houston, TX	7,627,942
IAD	Washington, DC	5,893,142
DFW	Dallas/Ft. Worth, TX	4,872,207
DTW	Detroit, MI	3,887,481
PHL	Philadelphia, PA	3,734,127
BOS	Boston, MA	3,673,748
FLL	Fort Lauderdale, FL	3,062,384
SEA	Seattle, WA	2,766,576

Data from [45].

### Influenza natural history and transmission

**Figure 1:**
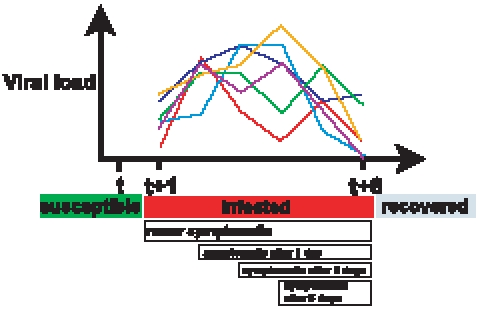
The natural history of influenza of simulated individuals in FLuTE. When a susceptible individual is infected (at time 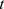), that person will be infectious for six days with infectiousness proportional to his or her viral load. The six possible viral load trajectories are plotted. Most individuals become symptomatic, which occurs after a 1, 2, or 3 day incubation period. Symptomatic individuals are twice as infectious as asymptomatic individuals (i.e., infectiousness is proportional to twice the viral load). Individuals recover six days after infection and are immune.

The current modeling of the natural history of influenza is as follows: An individual is infectious for six days starting the day after becoming infected. The individual's infectiousness is proportional to the log of the daily viral titers taken from a randomly chosen one of the six experimentally infected patients described in [12],[13] (Figure 1). An individual is asymptomatic during the incubation period, which lasts from one, two, or three days (with 30%, 50%, and 20% probabilities, respectively). After incubation, the individual has a 67% chance of becoming symptomatic [14],[15]. Symptomatic individuals are twice as infectious as asymptomatic people and may withdraw to the home after 0 to 2 days [16] (with probabilities summarized in Table 3). People who withdraw interact only with their households. Six days after infection, an individual recovers and is no longer susceptible.

**Table 3:** Probabilities that an individual will withdraw to the home 0, 1, or 2 days after becoming symptomatic.

*Age group*	0 days	1 day	2 days
Preschool-age children	0.304	0.575	0.324
School-age children	0.203	0.498	0.375
Adults	0.100	0.333	0.167

Data from [16].

The simulation runs in discrete time, with two time steps per simulated day to represent daytime and nighttime social interactions. The contact probability of two individuals in the same mixing group is the probability that they will have sufficient contact for transmission during a time step. Contact probabilities of individuals within families were tuned so that the simulated household secondary attack rates match estimates from [17] (Table 4). Contact probabilities within other mixing groups were tuned so that the final age-specific illness attack rates were similar to past influenza pandemics (Table 5), particularly Asian A (H2N2) and 2009 novel influenza A(H1N1) influenza, and the percentage of transmissions that can be attributed to each mixing group matched those in [6], [18]–[20], although these values depend on the transmissibility (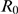) of the disease (Table 6). These contact probabilities are in general agreement with other simulation models [8] and with a recent study of physical contacts between individuals [21]. Contact probabilities for all types of mixing groups are summarized in Table 7.

**Table 4:** Estimates of secondary household attack rates from [17] and illness attack rates using FluTE, stratified by the ages of the index and secondary cases.

		*Exposed*			
		Addy 1991		simulated ( 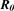 )	
		child	adult	child	adult
***Infectious***	**child**	29.0%	14.2%	28.6%	13.5%
	**adult**	10.3%	15.6%	9.3%	16.2%

**Table 5:** Age-specific influenza illness attack rates in past influenza epidemics (from [46]) and in a simulation of metropolitan Seattle.

Age group	Asian A (H2N2)	Hong Kong A (H3N2)	Age group	simulated
	1957–8	1968–9		( 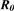 )
Pre-school children	35%	34%	0–4 years	38%
School-age children	55%	35%	5–18 years	53%
Young adults	25%	35%	19–29 years	26%
Middle adults	20%	32%	30–64 years	28%
Old adults	14%	31%	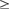 65 years	23%
Overall	31%	34%		33%

**Table 6:** Major sources of influenza transmission in simulations of metropolitan Seattle.

*Mixing group*	*Fraction of transmissions*		
	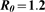	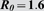	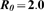
household	32%	31%	29%
schools/daycares	30%	24%	21%
workplace	10%	13%	15%
neighborhood/community	18%	21%	23%

**Table 7:** Person-to-person contact probabilities for all social mixing groups in FluTE.

	*Exposed*				
	*child 0–4*	*child 5–18*	*adult 19–29*	*adult 30–64*	*adult 65+*
*Family, infectious is child*	0.8	0.8	0.35	0.35	0.35
*Family, infectious is adult*	0.25	0.25	0.4	0.4	0.4
*Household cluster, infectious is child*	0.08	0.08	0.035	0.035	0.035
*Household cluster, infectious is adult*	0.025	0.025	0.04	0.04	0.04
*Neighborhood*	0.0000435	0.0001305	0.000348	0.000348	0.000696
*Community*	0.0000109	0.0000326	0.000087	0.000087	0.000174
*Workplace*			0.05	0.05	
*Playgroup*	0.28				
*Daycare*	0.12				
*Elementary school*		0.0348			
*Middle school*		0.03			
*High school*		0.0252			

**Figure 2:**
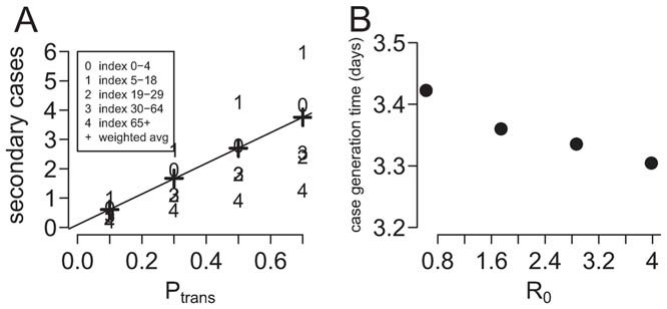
Influenza transmission properties in the simulation. (A) Observed secondary cases vs 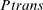 by the age of the index case and the weighted average. (B) Average case generation time vs 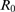.

Transmission probabilities in the simulation are adjusted by multiplying all contact probabilities by a scalar, 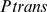, to obtain the desired 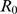, the basic reproductive number, which is defined as the average number of secondary infections from a typical infected individual in a fully susceptible population [22]. To derive the relationship between 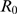 and 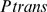, we infected a single randomly selected person in an otherwise fully susceptible 2000-person community with a 74% working-age adult employment rate and counted the number of individuals that person infected, repeating this procedure 1,000 times for several values of 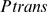. The relationship between 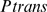 the average number of secondary cases was approximately linear for a biologically plausible range of values: 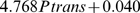 (Figure 2). However, the average number of secondary cases was higher when the index case was a child because children tend to infect more individuals (and become infected more often) than adults. Therefore, in a procedure borrowed from [6], we measured the age distribution of secondary cases when the index case was randomly selected and used this distribution to weight the contribution from the various age groups to the 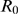 calculation to define 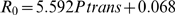. The definition of 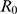 applies to a population with no pre-existing immunity, an assumption that may be violated for seasonal influenza. One can use the model to simulate seasonal influenza epidemics by substituting 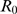 with the desired 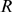, the average number of people a typical infected case infects in a population with pre-existing immunity.

The simulated case generation time, or the time between infection of an individual and the transmission to susceptibles, was 3.4 days for a wide range of 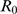 in a fully susceptible population (Figure 2B). This is consistent with other estimates for seasonal and pandemic influenza [20],[23].

### Simulated interventions

The primary pharmaceutical intervention is vaccination. Vaccinated individuals in the simulation have a reduced probability of becoming infected (VE*_S_*), of becoming ill given infection (VE*_P_*), and of transmitting infection (VE*_I_*) [24]. In the model, these efficacy parameters are implemented by multiplying the transmission probability per time step by (1−VE*_S_*) if the susceptible individual is vaccinated and by (1−VE*_I_*) if the infectious individual is vaccinated. The probability of vaccinated individuals becoming symptomatic (ill) after they are infected is the baseline probability (67%) multiplied by (1−VE*_P_*).

Vaccines do not reach full efficacy immediately – their protective effects may gradually increase over several weeks. The default behavior in the model is that the vaccine takes two weeks to reach maximum efficacy, with the efficacy increasing exponentially starting the day after the vaccination. Because of the delay in reaching maximum efficacy, it may be necessary to vaccinate the population early. In the simulation, vaccines can be administered at least four weeks before the epidemic (i.e., pre-vaccination), during the epidemic (reactive), or one dose can be administered at least three weeks before the epidemic and the boost can be administered reactively (prime-boost).

Antiviral agents (neuraminidase inhibitors) can be used for treatment of cases and for prophylaxis of susceptibles. A single course of antiviral agents is enough for 10 days of prophylaxis or 5 days of treatment. In the model, 5% of individuals taking antiviral agents prophylactically stop after 2 days and 5% taking them for treatment stop after 1 day [19]. As with vaccines, individuals taking antiviral agents can have reduced susceptibility (AVE*_S_*), probability of becoming ill given infection (AVE*_P_*), and transmitting infection (AVE*_I_*). However, unlike vaccines, the protective effects of the antiviral agents last only as long as they are being taken (5 to 10 days). When a case is ascertained, the individual is treated with antiviral agents, and that individual's household members will also each be given a course if household targeted antiviral prophylaxis (HHTAP) is in effect.

Several non-pharmaceutical interventions can be simulated in the model. *School closures* are simulated by eliminating school group contacts (including preschools and daycares but not playgroups) for those enrolled in school, but adding daytime contacts with other household members not in school or at work and doubling their daytime neighborhood and community contact probabilities to account for their non-school activities. Schools can be closed when cases are ascertained in communities or in the schools, and they can be closed for a fixed number of days or for the duration of the simulation.

During an epidemic, individuals may be requested to stay at home if they become ill. When simulating *isolation* of cases, individuals withdraw to the home one day after becoming symptomatic (with a certain probability to represent the compliance probability). This will eliminate any daytime social contacts that they have other than with household members who are not working or at school. We simulate a *liberal leave policy* in a similar manner: employed individuals withdraw to the home with a pre-set compliance probability for one week one day after becoming symptomatic.

During an epidemic, those living with symptomatic individuals may be requested to stay home [25]. In simulations of *household quarantine*, family members of symptomatic individuals will independently decide (based on a compliance probability) whether to obey quarantine for 7 days one day after the first individual becomes symptomatic. Individuals electing to quarantine themselves withdraw to the household and interact only with household members. If other family members become ill during quarantine, household members independently decide whether to obey quarantine for 7 days one day after each individual becomes symptomatic.

### Implementation of the stochastic model

FluTE is written in C/C++ and is released under the GNU General Public License (GPLv3, see http://www.gnu.org/licenses/gpl.html). The source code is available at http://www.csquid.org/software, https://www.epimodels.org/midas/flute.do, and the Models of Infectious Disease Agent Study (MIDAS) repository [26]. The software includes two source code files that are also freely distributable but may come with different licenses because they were written by others: one for the pseudorandom number generator (SIMD oriented Fast Mersenne Twister (SFMT) pseudorandom number generator [27]) and one to generate binomially distributed random numbers (from Numerical Recipes in C [28]). Version 1.11 of FluTE was used to produce the results in this manuscript.

A configuration file is used to specify the population to use for the simulation, the parameters for starting the epidemic, the transmissibility of the infectious agent, and the desired intervention strategies. The configuration file is text-based and can be typed in by a user or generated with a script. The simulation outputs results to text files, which can be easily parsed for plotting or statistical analysis.

A parallelized version of the code supports simulations of large populations (up to the entire continental United States). This version of the program assigns the populations of different counties to different processors, and OpenMPI is used to update the status of individuals who travel between communities that are located on different processors and to update the global status of the epidemic and the interventions (e.g., the total number of vaccines used). The simulation uses approximately 80 megabytes of memory per million simulated individuals.

The simulation was written with several competing goals: to explicitly represent each individual in the population, to conserve memory, to run quickly, and to be (relatively) easy to read and modify. Each simulated individual is represented by a C structure that includes unique identifiers for the person and for each of the social mixing groups to which that person belongs, the age of the individual, the person's infection and vaccination status and dates, and other attributes. For each infected individual, the simulation identifies all susceptible individuals in that person's community who share a common mixing group, the infectiousness of the infected individual, the susceptibility of the susceptible, and the probability that transmission takes place for every time step. Although comparing each individual with every other within a community results in the number of comparisons increasing with the square of the number of individuals, community sizes are always smaller than 3,000 residents. Therefore, the number of comparisons made between individuals scales approximately linearly with the number of individuals in the simulation. More sophisticated algorithms could improve the simulation's performance, but may do so at the expense of the code's flexibility and readability.

The running time depends on the number of individuals infected during the course of a simulation. Simulating an epidemic in a population of 10 million people can take up to two hours (on a single processor on an Intel Core2 Duo T9400), but it may take only seconds if the virus is not highly transmissible (low 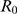) or if there are effective interventions (e.g., high vaccination rates). On a cluster of 32 processors, simulating an epidemic covering the continental United States (population of 280 million) takes about 6 hours (192 hours of total CPU time).

## Results

**Figure 3:**
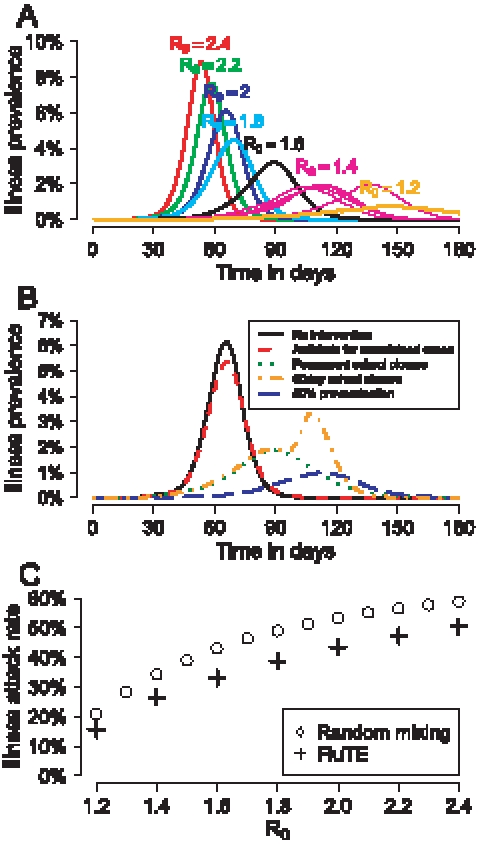
Illness attack rates and daily prevalence of influenza in simulations of metropolitan Seattle. (A) Daily prevalence of symptomatic influenza in simulations of metropolitan Seattle for various 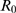 and (B) for 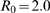 with various interventions. The interventions, which begin 30 days after the first case is detected, are: giving a course of antiviral agents to ascertained cases, closing schools either permanently or for 60 days, and pre-vaccination of 50% of the population with a well-matched seasonal influenza vaccine. (C) Final illness attack rates (180 days) vs 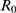 for FluTE (simulating metropolitan Seattle) and a model with random mixing. Results for all panels are from one run of metropolitan Seattle for each 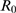 or intervention strategy except for the simulation for 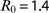 in panel (A), which was run 5 times with different random number seeds and plotted to show stochastic variability.

We illustrate the use of the model by simulating epidemics in metropolitan Seattle, a major metropolitan area with a population of approximately 560,000 according to the US 2000 Census. We ran simulations with different values of 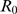, starting with ten infected individuals chosen at random, and found that the epidemic could peak as early as 45 days after the start if 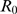 is high (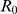) (Figure 3A). Pre-vaccination (with vaccine efficacies of VE*_S_* = 40%, VE*_P_* = 67%, VE*_I_* = 40%, which correspond to a well-matched seasonal influenza vaccine [29]) is likely to both lower and delay the epidemic peak (Figure 3B). Use of antivirals alone (AVE*_S_* = 30%, AVE*_P_* = 60%, and AVE*_I_* = 62% [11]) did not greatly reduce the epidemic peak, but they could reduce illness and mortality in an epidemic. Non-pharmaceutical interventions could be quite effective, but the epidemic may spike immediately upon ending the intervention (compare permanent school closure with school closure for 60 days in Figure 3B).

The illness attack rates in the simulation are lower than those in a SIR model with random mixing (where 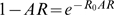 [30], where AR is the infection attack rate, and the illness attack rate is 0.67
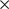
AR) (Figure 3C). As observed in earlier studies, models with community structure have lower attack rates than those with random mixing [31]–[33].

**Figure 4:**
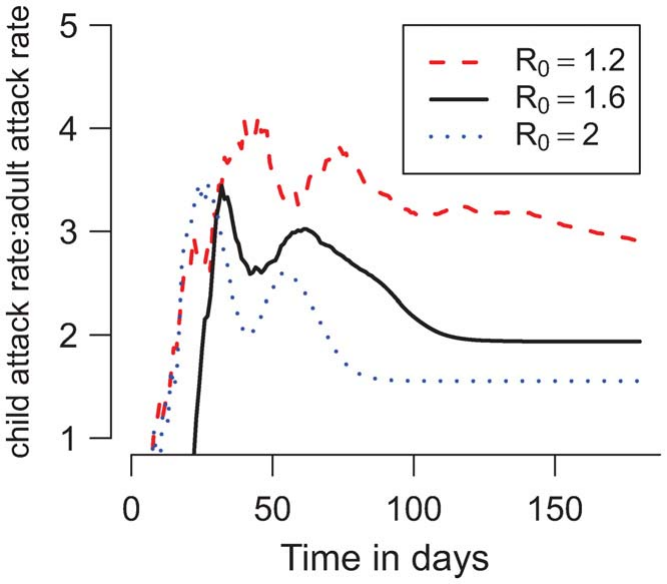
The ratio of cumulative illness attack rates between school-age children (ages 5–18) and adults (ages 19–64) over time in simulated epidemics. Results plotted are from one simulation of metropolitan Seattle for each value of 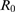.

Simulated epidemics struck school-age children earlier than adults, which had been observed in earlier studies [6],[34]. Therefore, we predict that early in an epidemic, the proportion of cases who are school-age children will be higher than later in the epidemic (Figure 4). This phenomenon might affect the accuracy of 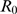 estimates in unfolding epidemics. For example, most confirmed cases in the recent novel influenza A(H1N1) outbreaks in the United States have been school-age children [35] and several early estimates of 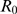 have been above 2 [36],[37]. In our model, we observed that infected children generate more secondary cases than infected adults (Figure 2A). For example, infected school-age children would transmit to an average of 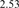 other individuals in a simulated epidemic with 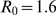. Therefore, estimates of 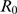 could be high early in an epidemic when a disproportionate number of infections are in children.

**Figure 5:**
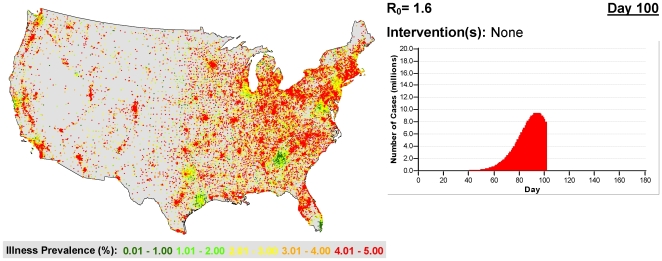
The prevalence of influenza in a single simulation of the United States 100 days after the start of an influenza epidemic with 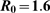. The color of each dot corresponds to the illness prevalence in a census tract. Image created using ArcGIS (Environmental Systems Research Institute, Inc.)

One can simulate the population of the entire continental US using the parallel version of FluTE (mpiflute). The continental US had 280 million people in 64735 census tracts in 2000, based on the US 2000 Census. In our simulations, we found that the final illness attack rates for the US to be nearly identical to those of metropolitan Seattle, but the epidemic peak for a given 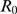 is later for the United States (e.g., 94 vs 65 days for 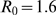) (Figure 5). Therefore, simulations of a sufficiently large metropolitan area may be adequate for determining the effect of a strategy on the national level on final illness attack rates, but the nation-wide peak of the epidemic may be later than in the major metropolitan areas because of the time it takes the epidemic to reach outlying areas.

## Discussion

We have described a new publicly available influenza epidemic simulator, FluTE. It explicitly represents every individual in the simulation, so simulated epidemics can be studied in detail, even tracing individual transmission events. We illustrated the use of FluTE with examples in which we explored the effect of various intervention strategies on influenza epidemics in the United States and showed how transmissibility can be over-estimated early in an epidemic.

The simulation was written so that one can easily set the transmissibility, vaccination policies (e.g., fraction of the population to vaccinate), and other reactive strategies (e.g., school closures). These settings can be used to investigate questions such as: 1) What fraction of the population will become infected or ill? 2) How much vaccine coverage is required to mitigate an epidemic with a given 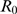
? 3) What segment of the population should be vaccinated to reduce overall illness attack rates the most? 4) How long can one wait before reacting to an epidemic? and 5) What range of 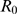 can be managed by a particular pandemic strategy? We have used FluTE to investigate some of these questions by simulating vaccinating children against seasonal and pandemic influenza [38] and pandemic mitigation [20].

The model was calibrated to simulate epidemics of a virus similar to 1957/1958 Asian A(H2N2) and 2009 pandemic A(H1N1). We attempted to model realistic pharmaceutical and non-pharmaceutical interventions, but their effects on an epidemic have not been well quantified. The model's results are plausible and likely to be qualitatively correct, but there is insufficient data to calibrate it to produce quantitatively accurate results for the various possible disease parameters and mitigation strategies. Although the model generates realistic population-level results, the spatial dynamics of the epidemics it produces should be used for illustrative purposes only. When using the model to evaluate mitigation strategies, it is important to consider one's goals. For example, using antiviral agents to treat cases does not greatly reduce the final illness attack rate in the simulation, but it could greatly reduce mortality. The model does not directly evaluate the cost of interventions, but the numbers of cases in a simulated epidemic can be linked to cost and healthcare utilization data [39].

Differential equation models are the most popular approach to disease modeling. The simplest of these (such as the SIR model [40]) can be used to study epidemics analytically, and more complex versions have been used to model the dynamics of epidemics on a global scale [41],[42]. However, if one wants to include a complicated natural history of disease or detailed intervention strategies, individual-based models, such as FluTE, may be more suitable.

The current software supports a limited set of configuration options and is intended for batch runs using a scripting language. Using the model for scenarios not supported by the existing code, such as testing a novel intervention strategy or altering the contact parameters for a different attack rate pattern, would require modification of the source code, which we have released so that others can make such changes if needed. We decided to adopt the GNU General Public License (GPL), so that the source code of derivative works must be released. We believe this will facilitate the sharing of improvements. The availability of source code allows others to adapt the model to simulate outbreaks of other airborne infectious diseases such as smallpox [3],[43],[44] or to simulate other regions of the world with different social structures [3].

In the future, we would like to make our model more accessible to non-programmers. This may involve developing a user interface or adding new parameters to the configuration file. We would also like to include intervention strategies that best reflect government pandemic mitigation plans. Achieving these goals would depend upon close collaboration with public health officials to better understand their needs and to carefully simulate existing pandemic mitigation plans and capacities. Although we have calibrated our model to the best available data, more detailed and reliable information on the natural history of influenza, influenza transmission, human behavior in response to infection, and vaccine efficacy is needed. Sensitivity analyses of similar epidemic models have shown that results are robust to uncertainty in many parameters [3],[5],[6],[11]. However, more accurate model inputs would improve the quantitative predictions. Well-designed studies are needed to acquire these data.
